# Acceptance and Adverse Effects of H1N1 Vaccinations Among a Cohort of National Guard Health Care Workers during the 2009 Hajj Season

**DOI:** 10.1186/1756-0500-4-61

**Published:** 2011-03-13

**Authors:** Gasmelseed Y Ahmed, Hanan H Balkhy, Saleh Bafaqeer, Badr Al-Jasir, Abdulhakeem Althaqafi

**Affiliations:** 1 King Abdullah International Medical Research Center, Riyadh, 11426, Saudi Arabia; 2 Department of Pediatrics, King Saud bin Abdulaziz University for Health Sciences, Riyadh, 11426, Saudi Arabia; 3 Infection Prevention and Control, King Abdulaziz Medical City, Riyadh, 11426, Saudi Arabia; 4 Gulf Cooperation Council (GCC) States and WHO Collaborating Center for Infection Prevention and Control, Riyadh, 11426, Saudi Arabia; 5 Department of Pediatrics, King Abdulaziz Medical City, Jeddah, 21423, Saudi Arabia; 6 Department of Family Medicine, King Abdulaziz Medical City, Jeddah, 21423, Saudi Arabia; 7 Infection Prevention and Control, King Abdulaziz Medical City, Jeddah, 21423, Saudi Arabia

## Abstract

**Background:**

The H1N1 influenza pandemic had garnered a large amount of attention. Currently, the most effective preventive measure available is the H1N1 vaccine. We aimed to assess the willingness of our study participants to receive the H1N1 vaccination prior to the annual Hajj season. If any participant declined, we investigated the reasons for vaccine rejection.

**Findings:**

We conducted a prospective cohort study of National Guard employees during the 1430 (2009) Hajj season. A survey was used as the primary method for data collection. Participants were vaccinated one to two weeks prior to their trip to Mona, and any side effects reported at the time of injection and three weeks post vaccination were recorded.

There were 100 male and 26 female participants in the study. In total, 66.7% (n = 84) of the participants were health care workers (HCWs) and 33.3% (n = 42) were non-health care workers (non-HCWs). Less than half of the respondents (46.8%, n = 59) accepted the vaccination. The vaccination acceptance rate was higher among non-HCWs, at a rate of 71.4% (n = 30); HCWs only accepted at a rate of 34.5% (n = 29) (OR 1.103, 95% CI [0.488-2.496]). The most common reason for vaccine refusal was the impression that the disease was not fatal (25.4%, n = 32). Finally, all participants reported pain at the injection site and 18.3% (n = 11) reported swelling. All other side effects were reported in less than 15% of the participants.

**Conclusions:**

Despite fears of the new H1N1 vaccine, there was a reasonable rate of vaccine acceptance among our study participants. Early health education may increase the rate of acceptance of the H1N1 vaccine. Furthermore, additional research is needed on long-term adverse effects of the H1N1 vaccine.

## Findings

**The Hajj **is an annual Muslim pilgrimage that begins on the first day of Dul-Hajja (12^th ^lunar month) and continues for 13 days. The Hajj occurs at the holy city of Mecca in the Kingdom of Saudi Arabia (KSA). The two- to three-million people who gather in the small, confined holy sites of the city come from multiethnic Muslim communities around the world [1-3].

**The Saudi National Guard **is a military and security force that includes several sub-administrations, of which one is the National Guard Health Affairs. This group is charged with providing health care services to National Guard personnel and their dependents. The group also offers medical services as necessary during the Hajj and other similar situations involving large groups of people.

Influenza pandemics are significant health threats that have occurred periodically over the past 300 years [4]. The most severe influenza pandemic, occurring from 1918 to 1919, killed approximately 30 to 50 million people worldwide. The emergence of the H5N1 avian influenza in 1997 promoted the recognition of the threat of another severe pandemic as a significant possibility, and such a pandemic would certainly be a threat at large gatherings such as the annual Hajj [5]. In response to this emerging threat, large-scale mitigation strategies have been developed. One cornerstone of these efforts is mass vaccination [6]. The current H1N1 pandemic, which was first recognized and defined in Mexico City in February 2009, spread rapidly throughout the world. Before the current Hajj season of 2009, the WHO declared there have been more than 300,000 laboratory confirmed cases of pandemic influenza H1N1 and 3,917 deaths in 191 countries [7].

In the current study, we assessed the acceptance of the H1N1 vaccination. We also examined possible barriers to vaccination within this high-risk cohort of employees, including both HCWs and non-HCWs, who worked at one of the largest human gatherings in the world.

## Materials and methods

### Setting

We conducted our study in Jeddah, the second-largest city in the KSA, where the National Guard medical and security teams were assigned to serve during the past Hajj season. Participants were recruited from the King Khalid National Guard Hospital, where the administration had assigned 150 HCWs and 350 non-HCWs for both health care and security services. This total number of employees was considered to be the baseline population for our study sample recruitment.

### Study design

The study was designed as a prospective cohort study. Participants were interviewed by the primary investigator after verbal consent was obtained. The first section of the interview included the collection of the following information: demographic characteristics, interest in vaccination, and reasons for refusing vaccination (if applicable). The second section of the interview focused on any experienced side effects (if applicable). The interview was conducted with all the employees staffed at the vaccination clinic two weeks prior to the beginning of the Hajj. After consenting to participate in the study, respondents were assessed for baseline measures, as well as signs and symptoms of the flu. After the assessment, willing participants were vaccinated. The cohort was followed during the Hajj for seven days and assessed one week after the end of the Hajj, a time when all employees had returned to their jobs in Jeddah City.

### Sample estimation

The sample size was estimated based on results from a previous study showing that 5% of participants declined the H1N1 vaccine [8]. Therefore, to determine an appropriate sample size for this study, we assumed that 10% of our population would refuse the vaccine. With a 5% margin for error and a power of 80%, these assumptions resulted in a necessary sample size of 199 participants. However, due to several logistic constraints, including delays in shipping by the manufacturing companies, only 126 participants (64% of the target sample size) were recruited.

### Selection of participants

All National Guard HCWs and non-HCWs assigned to serve during the Hajj season in the National Guard campus clinics were expected to visit the staff clinic to complete paperwork. Participants were recruited for the study two weeks prior to their travel to Mecca, and those who gave consent were interviewed. Inclusion criteria included being a National Guard employee and being assigned to serve during the 2009 Hajj season. Those with positive results on an H1N1 PCR-based assay during the current influenza season were excluded from the study.

### Instrument

The questionnaire was written in English, translated into Arabic, and back-translated into English. It was reviewed by an experienced bilingual medical research staff member for content validity. Both forms were reviewed and approved by the Institutional Review Board of the King Abdullah International Medical Research Center (IRB of KAIMRC). The questionnaire contained 30 questions that focused on identifying information and socio-demographic characteristics. Those who accepted the vaccine were followed-up and questioned about immediate side effects. Those who refused the vaccine were questioned about their reasons for declining and about the most effective preventive measures they would apply in place of vaccination.

### Interviews and data collection

All interviews were conducted during the employees' visit to the staff clinic, which was two weeks prior to arriving at Mecca. The follow-up lasted for a one-week period that followed the employees' work during the Hajj. A final follow-up visit for each participant occurred no less than ten days after the end of the Hajj. All questions were administered by a single interviewer to reduce possible bias. Each interview lasted for ten minutes, and the interviewer reviewed the questions for completeness after the end of each interview.

### Vaccine

The Pandemrix H1N1 vaccine was administered during the study. Each 0.5-ml vaccine was injected intramuscularly into the upper arm. This medication is a split inactivated influenza virus vaccine containing antigen equivalent to the A/California/7/2009(H1N1)v-like strain (X-179A). Each 0.5-ml dose contained 3.75 μg of egg-propagated hemagglutinin-containing immunologic adjuvant [9].

### Statistical data analysis

Data entry was performed using Microsoft Excel. After entry, the data were transferred to SPSS version 17 software for detailed descriptive analysis. The means and standard deviations of age were calculated.

## Results

### Demographic characteristics of study participants

A total of 126 of 500 assigned employees were recruited for the study (25.2%). Of the total study participants, 100 (79.4%) were male and 26 (20.6%) female, with a mean age of 38.7 and standard deviation of 9.8 years. The majority of participants (n = 67, 53.2%) refused the H1N1 vaccination and preferred using other protective measures. The participants who were non-HCWs had a vaccine acceptance rate of 71.4%, whereas HCWs had a vaccine acceptance rate of 34.5% (OR of 1.103, 95% CI [0.488-2.496]).

### Vaccine acceptance and preventive measures for disease transmission

Table 1 shows that 27 (21.4%) of the participants who refused vaccination cited safety as the major reason for refusal. There were 29 (23.0%) participants who cited the presence of toxic preservatives in the vaccine (adjuvant and mercury) as their major reason for refusal. Additionally, 25 (19.9%) participants cited the difference of the vaccine from the one provided to western communities, which does not contain an adjuvant or mercury. There were 26 (20.6%) participants who reported having received confusing information from the media and the government; they blamed the media for misleading the public about the spread of the disease, its fatality, and the long- and short-term toxic effects of the vaccine. In addition, 32 (25.4%) participants claimed that the swine flu was not a fatal disease and that becoming infected was preferable to receiving the vaccination. Furthermore, 31 (24.6%) participants thought that their environment was not optimal for a swine flu outbreak. Two participants stated that they would have accepted the vaccine had it been available as a nasal spray. Additionally, four HCWs claimed that they had already been exposed to infected patients during their daily clinical practice and had become carriers; therefore, they believed that they probably already had an acquired immunity. Other participants preferred applying alternative protective measures in lieu of accepting the vaccination. Of these participants, 19 (15.8%) preferred to eat honey, and 25 (19.8%) preferred to eat citrus fruits as immunity strengtheners. All participants reported frequent hand washing, and 63 (50.0%) also reported wearing a mask as an additional protective measure.

**Table 1:** Sociodemographic characteristics of National Guard employees assigned to serve during the 2009 Hajj season and reasons for refusing the H1N1 vaccination.

Reasons for refusal	Refusing n (%)	Sex n (%)		Employee status n (%)	
		**Male** **n = 100**	**Female** **n = 26**	**HCWs** **n = 42**	**Non-HCWs** **n = 84**

No adequate safety studies	27 (21.4%)	20 (15.9%)	7 (5.5%)	2 (1.6%)	25 (19.8%)
Contains toxic preservatives	29 (23.0%)	21 (17.0%)	8 (06%)	3 (2.4%)	26 (20.6%)
Different from western vaccine	25 (19.9%)	18 (14.4%)	7 (5.5%)	1 (0.9%)	24 (19.0%)
Misleading media information	26 (20.6%)	23 (18.3%)	3 (2.3%)	12 (09.5%)	14 (11.1%)
Swine flu not a fatal disease	32 (25.4%)	25 (19.9%)	7 (5.5%)	7 (5.5%)	25 (19.9%)
This is not an environment for H1N1	31 (24.6%)	25 (19.9%)	6 (4.7%)	9 (7.1%)	22 (17.5%)
Frequent hand washing	126 (100%)	100 (79.4%)	26 (20.6%)	36 (28.6%)	90 (71.4%)
Wearing a mask	63 (50.0%)	48 (38.1%)	15 (11.9%)	13 (10.3%)	50 (39.7%)
Eating honey	19 (15.8%)	14 (11.2%)	05 (04.6%)	7 (05.5%)	12 (10.3%)
Eating citrus	25 (19.8%)	21 (16.7%)	4 (03.2%)	6 (04.7%)	19 (15.1%)

### Adverse vaccine events

The adverse events listed in Table 2 show that 100% of participants reported feeling localized pain and soreness, and 11 (18.3%) reported minor local swelling at the site of injection. Headache and body aches were reported in 8 (13.4%) of the participants, while 20 (33.3%) participants reported feeling fatigue and malaise. All other adverse events occurred in less than 15% of the participants, Table 1.

**Table 2:** Common adverse effects of H1N1 vaccination among National Guard employees assigned to serve during the 2009 Hajj season.

Adverse Effects	Present	Sex	
	Total Number (%)	Male Number (%)	Female Number (%)
Pain at injection site	59 (100%)	46 (78.1%)	13 (21.9%)
Swelling at injection site	11 (18.3%)	09 (15.3%)	2 (3.0%)
Headache	8 (13.4%)	06 (10.2%)	2 (3.2%)
Body ache	8 (13.4%)	07 (11.9%)	1 (1.5%)
Fatigue	20 (33.3%)	17 (28.8%)	3 (4.5%)
Fever	3 (5.1%)	3 (5.1%)	0
Vomiting	2 (3.4%)	2 (3.4%)	0
Flu-like illness	3 (5.1%)	3 (5.1%)	0
Dizziness	1 (1.7%)	1 (1.7%)	0
Numbness	1 (1.7%)	1 (1.7%)	0

### Follow-up period

**Figure 1:**
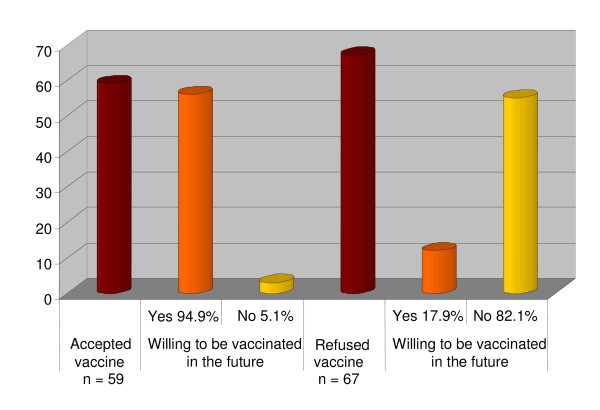
**Willingness to be vaccinated in the future among National Guard employees serving in the Hajj who either declined or accepted the H1N1 vaccine (n = 126)**.

At the end of the follow-up period, which was approximately three weeks after receiving the vaccination, all participants were interviewed about whether they would be willing to receive the same vaccination if they were approached the following year. Overall, 94.9% of those vaccinated answered positively, whereas only 17.9% of those who refused vaccination answered positively. The main explanation from the latter group was that they had not experienced any of the adverse effects that were experienced by their colleagues following the vaccination. The remaining 82.1% of those who refused vaccination stated that they were still worried about its long-term adverse effects. The distribution of responses is illustrated in Figure 1.

## Discussion

In 2009, the large gathering of the annual pilgrimage (known as the Hajj) occurred concomitantly with the swine flu pandemic and the global panic surrounding the possibility of a rapid outbreak. For these reasons, we were interested in studying high-risk participants who were assigned to provide services during the pilgrimage. This allowed us to gather information about their attitudes toward the H1N1 vaccine. We were also interested in identifying and assessing the short-term adverse effects of the newly manufactured vaccine [10]. Regarding the current pandemic, the WHO had recommended that all countries give high priority to immunizing their HCWs to protect the essential health infrastructure. Despite these recommendations, the acceptance of voluntary vaccination was unexpectedly low [11].

This study explored reasons for swine flu vaccination refusal during a time of global public concern and anticipation of a global outbreak. In our cohort of Saudi National Guard employees assigned to work during the Hajj, 46.8% of participants accepted the vaccination. We found that more non-HCWs (mainly military personnel) accepted the vaccine than HCWs. This was consistent with our previously published finding that physicians had a low acceptance rate for both seasonal and pandemic vaccinations in early 2009/2010 [12].

Despite strong recommendations to vaccinate healthcare workers with the influenza vaccine, coverage was exceedingly low for all specialties, with some differences according to location and type of employment [13]. In addition to education about preventive measures, such as frequent hand washing and wearing a mask, vaccination is an essential protective measure. Influenza vaccination among healthy working adults has been shown to be highly effective, resulting in a 25% reduction of upper respiratory illness [14]. In this study, the reasons for refusal included worries about vaccination safety and doubts about vaccine efficacy. In a study from Hong Kong, the influenza vaccine was efficient in preventing H1N1 infection in 61% of the participants [15]. In addition, some participants believed that other preventive measures could be applied to yield the same benefits; misleading information from the media may have been the source of this reasoning. One of the prominent sources of concern was the difference in chemical constituents between the vaccine used in developed countries and those used in developing countries including the Kingdom. Participants' hesitance, worry, and safety concerns about using a newly developed vaccine were other major determinants of negative response and vaccination refusal. The most common reasons for accepting the vaccination were a "wish to be protected" and "following health authority advice." The most common reason for refusal was "worry about side effects." In addition, some other reasons for refusing vaccination included "doubts about the efficacy of the vaccine," feeling that it was "not yet the right time to be vaccinated," and "simply not wanting the vaccine [15]." Vaccine-seeking consumers must first be convinced of a reasonable likelihood that the disease will occur in their location and that they are susceptible. Additionally, they must be convinced that the disease is serious. Finally, they must be convinced of the safety, if not the efficacy, of the vaccine before they will accept it [15]. In a recent study conducted on a group of Saudi civilians, we were able to confirm that the media played a major role in decreasing the acceptance of the vaccine. Furthermore, the lack of public education by knowledgeable HCWs may have also contributed to popular belief in the negative propaganda [16]. Campaigns and health education in advance to the next anticipated influenza outbreaks could play an essential role in encouraging communities to accept vaccinations during similar future circumstances.

Side effects experienced by the vaccinated individuals in the cohort consisted of normal short-term adverse effects [17]. Therefore, the vaccine is promising for future use if any outbreak is expected, assuming that further assessment of the long-term adverse effects yields positive results.

Based on surveillance following the 1976 swine flu vaccination program, the risk of anaphylaxis from the influenza vaccination was estimated to be approximately one in every four million people. In 1976, Guillain-Barré syndrome was associated with receipt of the swine flu vaccine, with a risk of 1 per 100,000 individuals vaccinated [18]. Safety and efficacy are critical factors in determining the rate of vaccination in the general population. Governments that want to promote H1N1 vaccination will need to gain a better understanding of the barriers to and facilitators of acceptability before implementing full-scale vaccination programs [19].

The current study demonstrated a substantial increase in participant awareness. Most participants understood that applying as many protective measures as possible allows for the best protection from swine flu infection. Our results also suggest that frequent hand washing is seen as one of the most influential protective measure. When participants were asked about future vaccines three weeks after the vaccination campaign, an increased number of participants (11 out of 67) had changed their minds and would accept vaccination in the future. This change of opinion among the participants could contribute to a higher acceptance rate if vaccinations are offered during future pandemics. The presence of participants in the same campus for three weeks post-vaccination, coupled with an exchange of information between the accepting and refusing groups, may have been the reasons that some participants changed their willingness to receive a future vaccination. HCWs were seriously deficient in terms of their knowledge of influenza prevention. Extensive and sustained efforts to overcome these limitations are urgently needed among HCWs, regardless of whether they are involved in direct or indirect patient care [20]. These efforts will help to increase effective compliance among HCWs and the general public.

## Limitations

Several limitations of this study were considered and acknowledged by our research team. First, the inclusion of a specific cohort of National Guard employees limits the wider generalizability and application of the research findings, as these employees are mainly from the local area and have extensive experience with the Hajj. Therefore, our cohort may not feel threatened by participation in such an international mass gathering. Second, the small sample size of participants may have hindered the ability to detect statistical significance; this may have resulted in a type II error (i.e., the sample size was not sufficiently large to confirm a difference between the groups).

## Conclusions

In the context of global panic surrounding H1N1 and the possibility of an outbreak in a large gathering such as the Hajj, this study found that the acceptance rate for mass vaccination was low among HCWs but acceptable among non-HCWs. Further, our study recognized and characterized major barriers preventing H1N1 vaccination acceptance. Another significant finding of our analysis was the report of mild (within the normal range) short-term adverse effects experienced by our cohort.

## Competing interests

The authors declare that they have no competing interests.

## Authors' contributions

GYA and HHB were involved in the design and preparation of the research proposal. GYA was involved in the direct patient interviews, data collection, data analysis, and manuscript preparation. HHB was involved in the final revision of the manuscript. All authors read and approved the research proposal and final manuscript.
